# Tumor necrosis factor-α blockade in recurrent and disabling chronic sciatica associated with post-operative peridural lumbar fibrosis: results of a double-blind, placebo randomized controlled study

**DOI:** 10.1186/s13075-015-0838-4

**Published:** 2015-11-19

**Authors:** Christelle Nguyen, Clémence Palazzo, Sophie Grabar, Antoine Feydy, Katherine Sanchez, Nathalie Zee, Laurent Quinquis, Myriam Ben Boutieb, Michel Revel, Marie-Martine Lefèvre-Colau, Serge Poiraudeau, François Rannou

**Affiliations:** Univ. Paris Descartes, PRES Sorbonne Paris Cité, Service de Rééducation et Réadaptation de l’Appareil Locomoteur et des Pathologies du Rachis, Hôpital Cochin, Assistance Publique - Hôpitaux de Paris, Paris, France; Univ. Paris Descartes, PRES Sorbonne Paris, Cité Laboratoire de Pharmacologie, Toxicologie et Signalisation Cellulaire, INSERM UMR-S 1124, UFR Biomédicale des Saints Pères, Paris, France; Univ. Paris Descartes, PRES Sorbonne Paris, INSERM UMR-S 1153 et Institut Fédératif de Recherche sur le Handicap, Paris, France; Univ. Paris Descartes, PRES Sorbonne Paris, Biostatistics and Epidemiology Unit, Hôtel Dieu, Assistance Publique-Hôpitaux de Paris, Paris, France; INSERM UMR-S 1136, Institut Pierre Louis d’Épidémiologie et de Santé Publique, F-75013, Paris, France; Univ. Paris Descartes, PRES Sorbonne Paris Cité, Service de Radiologie B, Hôpital Cochin, Assistance Publique - Hôpitaux de Paris, Paris, France

**Keywords:** TNF-α blockade, Infliximab, Sciatica, Post-operative lumbar peridural fibrosis, Randomized controlled trial

## Abstract

**Introduction:**

The aim of this study was to assess the efficacy and safety of tumor necrosis factor (TNF)-α inhibition with infliximab (IFX) in treating recurrent and disabling chronic sciatica pain associated with post-operative peridural lumbar fibrosis.

**Method:**

A double-blind, placebo-controlled study randomized 35 patients presenting with sciatica pain associated with post-operative peridural lumbar fibrosis to two groups: IFX (n = 18), a single intravenous injection of 3 mg/kg IFX; and placebo (n = 17), a single saline serum injection. The primary outcome was a 50 % reduction in sciatica pain on a visual analog scale (VAS) at day 10. Secondary outcomes were radicular and lumbar VAS pain at day 0 and radicular and lumbar VAS pain, Québec disability score, drug-sparing effect and tolerance at days 10, 30, 90, and 180.

**Results:**

At day 10, the placebo and IFX groups did not differ in the primary outcome (50 % reduction in sciatica pain observed in three (17.6 %) versus five (27.8 %) patients; *p* = 0.69). The number of patients reaching the patient acceptable symptom state for radicular pain was significantly higher in the placebo than IFX group after injection (12 (70.6 %) versus five (27.8 %) patients; *p* = 0.01). The two groups were comparable for all other secondary outcomes.

**Conclusion:**

Treatment with a single 3 mg/kg IFX injection for post-operative peridural lumbar fibrosis-associated sciatica pain does not significantly reduce radicular symptoms at day 10 after injection.

**Trial registration:**

ClinicalTrials.gov NCT00385086; registered 4 October 2006 (last updated 15 October 2015).

## Introduction

The prevalence of persistent or recurrent post-operative back or lower limb pain in patients who undergo discectomy or laminectomy is up to 15 % [1], and management remains challenging. Recurrent symptoms in the legs are thought to be related to recurrent disc herniation, persistent herniated fragment, spinal stenosis, or post-operative peridural fibrosis secondary to scar formation [2, 3]. Extended peridural fibrosis is associated with poor surgical outcomes in 24 % of patients after disc herniation surgery [3]. Peridural scarring is consistently observed after spinal surgery including discectomy [4, 5] or laminectomy [6, 7]. However, with extended and adhesive post-operative peridural fibrosis being associated with inflammatory changes at the surgical site, nerve root and dural sac neuromechanics are impaired, which ultimately leads to nerve root compression, abnormal dura and nerve root bounding and traction during back and limb movements [1, 8]. Peridural fibrosis may also impair the diffusion of nutrients causing nerve root starvation [9]. Patients with post-operative peridural lumbar fibrosis-associated sciatica pain often experience loss of function, disability and impaired quality of life and can require the use of opioid analgesics [10]. Etiologic diagnosis of recurrent or persistent post-operative sciatica is currently based on magnetic resonance imaging (MRI), which helps distinguish central and lateral spinal stenosis from disc herniation, persistent herniated fragment or peridural fibrosis [11]. In the case of peridural fibrosis, MRI demonstrates hyposignals in T1-weighted sequences with enhancement after gadolinium injection and hypersignals in T2-weighted sequences of the fibrotic tissue in the peridural space.

Key and Ford first demonstrated that experimental destruction of the intervertebral disc peripheral annulus fibrosus resulted in peridural scarring [4]. Three factors causing scar formation were peridural fat destruction, peridural hematoma and invasion of the surgical trajectory by paravertebral muscle fibers [7]. Treatments targeting one of these three factors included: interposition of biological material such as fat and animal collagen fibers [12–16]; nonbiological material such as absorbing gelatin sponge, silastic membrane, wax for bone hemostasis or polyglactine 910 [7, 13, 15, 17–19]; and viscous material such as carboxymethylcellulosis or sodium hyaluronate [15, 20–22], with only limited efficacy. Radiotherapy [23, 24] and treatment with human recombinant interferon-γ [25] and D-penicillamine [26, 27] also demonstrated little efficacy. Surgery aiming to remove peridural fibrosis was abandoned because of poor mid- and long-term outcomes, and the high incidence of adverse events [2, 28–31]. The most frequently used treatment is intradural [32] or peridural corticosteroid injections [33]. However, controlled trials addressing their efficacy in the setting of sciatica pain associated with post-operative peridural lumbar fibrosis are lacking. Only one randomized controlled study compared the efficacy of forceful peridural corticosteroid injections via the sacral hiatus to simple peridural corticosteroid injections on sciatica pain ascribed to post-operative lumbar spinal fibrosis. At 6-month follow-up, the proportion of patients with sciatica relief was significantly higher with forceful than simple injection (29 (45 %) versus 31 (19 %); *p* = 0.03) [34].

Recently, cytokine inhibitors have generated intense interest as a possible treatment for radiculopathy. The inflammatory cytokine tumor necrosis factor (TNF)-α promotes the production of pro-inflammatory soluble mediators, angiogenic factors and chemokines by various cell types and tissues [35]. TNF-α also promotes tissue fibrosis by stimulating fibroblast proliferation and modulating their chemotaxis [36]. In addition, TNF-α blockade with anti-TNF-α monoclonal antibodies has demonstrated some efficacy in conditions associated with tissue fibrosis [37]. In murine models of lung fibrosis induced by bleomycin or silicium oxide particles, the TNF-α level was increased in lung tissue. TNF-α injection induced changes mimicking those observed in lung fibrosis, such as increased number of fibroblasts, collagen deposits and necrosis. Conversely, treatment with TNF-α blockers significantly reduced lung fibrosis [38, 39]. Consistently, TNF-α played an important role in a porcine bronchial model of obliterative bronchiolitis with fibrosis, and its blockade was associated with decreased lesions [40]. Most recently, topical application of etanercept, a TNF-α blocker, was effective in reducing epidural fibrosis in rats after laminectomy [41].

Altogether, clinical and experimental data suggest a pathogenic role of TNF-α in scar formation and fibrotic processes. We hypothesized that blocking TNF-α may be of interest in management of post-operative peridural lumbar fibrosis, and we aimed to assess the efficacy and safety of TNF-α inhibition with infliximab (IFX) on the associated sciatica pain 10 days after the treatment.

## Methods

### Design

We conducted a parallel-group, double-blind, randomized placebo-controlled monocentric study in a tertiary care hospital (Cochin Hospital, Paris, France).

### Patient selection

Patients referred to our rehabilitation department for recurrent sciatica after discectomy were screened. Inclusion criteria were age >18 years old, sciatica post-discectomy, radicular pain measured on a visual analog scale (VAS) >40 mm and inability to perform usual activities, surgical discectomy (between 2 years and 6 months previously), pain-free between 1 month and 1 year after the discectomy, MRI with gadolinium injection <6 months and performed >6 months after the discectomy, presence of peridural fibrosis on MRI, and failure of peridural injection treatment. Exclusion criteria were untreated chronic psychiatric disorders, presence of a conflict between the nerve root and herniated disc or disc fragments or spinal stenosis, severe cognitive impairment, inability to understand and speak French, enrollment in another clinical trial in the previous 3 months, and contraindications to IFX treatment including previous allergic reactions to IFX or its components, classes III or IV cardiac failure, active or latent tuberculosis evidenced by clinical examination, tuberculin intra-dermal reaction and chest X-ray, severe infections, pregnancy, breastfeeding, absence of contraception for women, and cancer <5 years.

### Patient characteristics at baseline

The following parameters were recorded for each patient at baseline: age; sex; height; weight; retirement and sick leave status; lumbar, radicular and neuropathic pain on a VAS (0–100 mm); Québec disability score (20 items; scored from 0 = no disability to 5 = impossible to do; final score 0–100); self-reported clinically significant symptoms of anxiety or depression by the Hospital Anxiety and Depression Scale (HAD-S; seven items related to anxiety and seven to depression; each item scored on a scale from 0 to 3; total score ranging from 0 = no depression, no anxiety to 21 = maximal depression, maximal anxiety), the Fear-Avoidance Beliefs Questionnaire score (FAB-Q; five items related to physical activity and seven to work; each item scored on a scale from 0 = “do not at all agree” to 6 = “completely agree”; total score ranging from 0 = low fear-avoidance beliefs to 24 for the physical activity dimension or 42 for the work dimension, maximal fear-avoidance beliefs); number of lumbar surgeries; time between surgery and recurrent radicular pain; time between surgery and inclusion; and co-interventions.

### MRI examination

All patients had a lumbar MRI with T1, T2 and T1 with gadolinium injection sequences. Lumbar MRI findings were examined independently by a blinded assessor at baseline for the following parameters: presence or absence of a retractile scar, nerve root enhancement, nerve root enlargement, arachnoiditis, and Modic 1 vertebral endplate subchondral bone changes.

### Intervention

The IFX group received a single intravenous infusion of 3 mg/kg IFX (REMICADE, Schering-Plough Centocor, B.V., AMM n° EU/1/99/116/001; CIP 562 070.1) over a 2-hour period, with vital signs assessed by a physician for the first 10 minutes, then by a nurse for the rest of the infusion time and for an additional 2 hours. The placebo group received a single saline serum infusion (9 % sodium chloride, Maco Pharma) over a 2-hour period, with vital signs assessed by a physician for the first 10 minutes, then by a nurse for the rest of the infusion time and for an additional 2 hours. Co-interventions were allowed and recorded. Unauthorized co-interventions were immunosuppressants.

### Outcome measures

The primary outcome was 50 % reduction in sciatica pain measured on a VAS (0–100 mm) at day 10. Secondary outcomes were sciatica pain assessed by a VAS at 2 hours and days 30, 90 and 180; lumbar pain assessed by a VAS at 2 hour sand days 10, 30, 90 and 180; patient acceptable symptom state (PASS) defined as a VAS score <40 mm; and absolute and relative minimum clinically important improvement (MCII) defined as a reduction in VAS-assessed pain ≥15 mm or ≥20 %, respectively, at 2 hours and day 10; Québec disability score; co-interventions; and tolerance at days 0, 10, 30, 90 and 180.

### Tolerance

Adverse effects during infusion or during follow-up, such as anaphylaxis, fatigue, chest pain, dyspnea, headache, vertigo, dizziness, abdominal pain, nausea, diarrhea, dyspepsia, liver test abnormalities, rash, flush, pruritus, urticaria, skin dryness, increased sweating, respiratory tract infections, sinusitis, and viral infection were recorded. In the case of a reaction to infusion, infusion speed was reduced.

### Sample size

To achieve a 50 % reduction in sciatica VAS pain score between IFX and placebo groups, with an α risk of 0.05, a power (1-β) of 0.80, and predicted improvement in pain scores of 10 % in the placebo group and 60 % in the IFX group, the number of participants needed was 17 in each group (two-sided chi-square test). With an estimated 10 % of patients lost to follow-up or presenting latent or active tuberculosis, we sought to include 20 patients in each group.

### Randomization and allocation concealment

Patients who met the inclusion criteria and agreed to participate were randomly assigned to the IFX or placebo group. The randomization process was centralized at the coordinating office (Unité de Recherche Clinique, Cochin Hospital), which had no involvement in the enrollment, follow-up, or assessment of participants. A statistician produced a computer-generated randomization list at the coordinating office with block size of 4. Once the screening process was complete, the investigator sent a fax to the coordinating office. The coordinating office randomly assigned the patient to a treatment and faxed the investigator the allocated treatment. IFX and placebo doses were prepared by the pharmacy of the hospital according to the randomization list.

### Blinding

Patients and assessors were blinded to the treatment assigned. Presentations, treatment administration and clinical monitoring of the treatments were strictly identical for each patient. Labeling was anonymized by the pharmacy.

### Statistical analysis

Data analysis involved the use of SAS 9.3 (SAS Institute, Cary, NC, USA). Blinded statisticians (MBB, SG and LQ) performed the statistical analyses at an independent center (Unité de Biostatistique et d’Épidémiologie, Hôtel Dieu, GH Cochin). All analyses were performed on an intent-to-treat basis, in that all patients were considered in the analysis and were analyzed in the group to which they had been assigned. Treatment of missing data involved the last-observation-carried-forward method. For descriptive analyses, qualitative variables are reported with absolute and relative frequencies, and quantitative variables with median (interquartile range (IQR)). For comparative analysis, qualitative variables were compared by chi-square test or Fisher’s exact test in cases of low frequency of the observed event. Quantitative variables were compared by Student *t* test or nonparametric Wilcoxon Mann-Whitney test if the samples were insufficient. VAS at day 10 was also compared quantitatively using an analysis of covariance (ANCOVA) to control for baseline VAS measure imbalanced between treatment groups.

### Ethics approval

In accordance with L.1123-6 article of the French Health Code, the study protocol was submitted and approved by the local ethics committee (Comité consultatif de Protection des Personnes en Recherche Biomédicale de l’Île-de-France). All patients gave written informed consent to participate.

### Role of the funding source

The Assistance Publique-Hôpitaux de Paris (Project no. P050312) funded the study. The funding source was not involved in the design or conduct of the study or collection, management, and analysis of the data. It was not involved in the writing or final approval of the manuscript. Authors did not receive compensation or funding for conducting independent data analyses. The corresponding author had full access to all the data in the study and takes responsibility for the integrity of the data and the accuracy of the data analysis.

## Results

### Patient recruitment

**Fig. 1:**
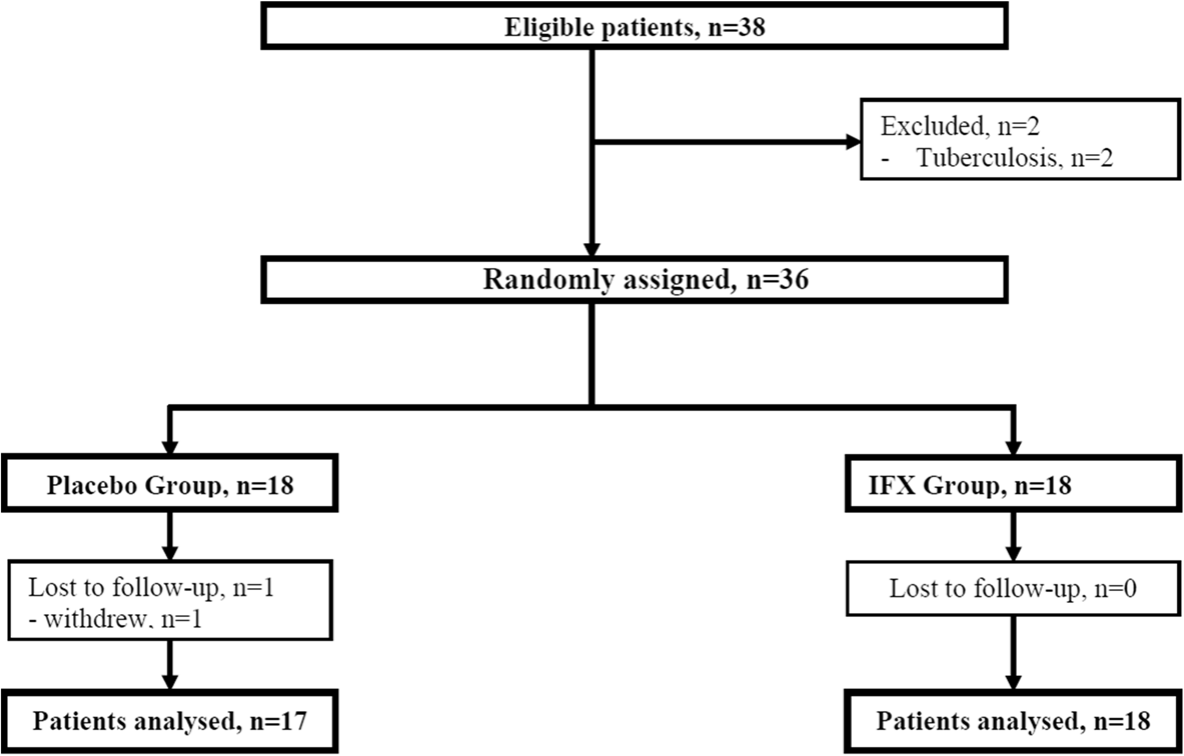
Flow of patients in the trial. *IFX* Infliximab

In total, 38 patients met the inclusion criteria; two were excluded because of evidence of tuberculosis during the screening. From February 2007 to December 2011, we randomly assigned 18 patients to the placebo group and 18 to the IFX group. One patient was lost to follow-up in the placebo group (withdrew after randomization and before treatment) and none in the IFX group. Overall, data were available for analysis for 17 patients in the placebo group and 18 in the IFX group (Fig. 1).

### Baseline characteristics of patients

The median age was 44.0 years (range 38.0–8.3 years), and the male:female ratio was 1:1; 31 patients (88.6 %) were on sick leave at the time of inclusion (Table 1). The median Québec score was 48.0 (37.0–63.0). Patients underwent from one to four lumbar surgeries before inclusion, with a median (IQR) time between surgery and recurrent radicular pain of 92.0 (61.0–153.0) days, and a median (IQR) time between the last surgery and inclusion of 2.3 (1.6–3.6) years. Co-interventions included analgesics, nonsteroidal anti-inflammatory drugs, corticosteroids, antidepressants, anxiolytics and antiepileptics, and were found in 30 patients (85.7 %). The most frequent MRI lumbar feature was nerve-root enhancement, seen in 25 patients (71.4 %), followed by presence of a retractile scar in 23 (65.7 %). Modic 1 vertebral endplate subchondral bone changes detected by MRI were present in 19 patients (54.3 %) in total.

**Table 1:** Patient demographics, low back pain characteristics, and MRI features at baseline

	IFX group	Placebo group	All patients
	n = 18	n = 17	n = 35
Age (years), median (IQR)	45.0 (38.0–52.0)	43.0 (40.0–48.0)	44.0 (38.0–8.3)
Male sex, n (%)	11 (61.1)	7 (41.2)	18 (51.4)
BMI (kg/m^2^), median (IQR)	27.9 (24.8–29.8)	25.8 (23.0–29.0)	26.9 (24.4–29.8)
Retired, n (%)	0 (0)	0 (0)	0 (0)
Sick leave, n (%)	16 (88.9)	15 (88.2)	31 (88.6)
Lumbar pain (VAS (0–100 mm)), median (IQR)	60.0 (41.0–80.0)	50.0 (26.0–60.0)	58.0 (35.0–70.0)
Radicular pain, (VAS (0–100 mm)), median (IQR)	70.0 (65.0–85.0)	55.0 (50.0–70.0)	65.0 (45.0–76.0)
Neuropathic pain, n (%)	18 (100)	11 (64.7)	29 (82.9)
Neuropathic pain (VAS (0–100 mm)), median (IQR)	62.5 (40.0–75.0)	45.0 (41.0–75.0)	50.0 (41.0–70.0)
Québec score (0–100), median (IQR)	55.0 (37.0–68.0)	45.0 (31.0–62.0)	48.0 (37.0–63.0)
HAD-S, median (IQR)			
HAD anxiety score (0–21)	10.0 (6.0–15.0)	10.0 (8.0–14.0)	10.0 (6.0–14.0)
HAD depression score (0–21)	9.0 (6.0–11.0)	7.0 (5.0–9.0)	7.5 (6.0–11.0)
FAB-Q, median (IQR)			
FAB-Q work subscale score (0–42)	33.0 (21.0–41.0)	24.0 (19.0–35.0)	30.0 (20.0–39.0)
FAB-Q physical activity subscale score (0–24)	14.0 (11.0–19.0)	16.0 (10.0–19.0)	14.0 (10.0–20.0)
Number of lumbar surgeries, n (%)			
1	9 (50.0)	4 (23.5)	13 (37.1)
2	3 (16.7)	9 (52.9)	12 (34.3)
3	4 (22.2)	3 (17.7)	7 (20.0)
4	2 (11.1)	1 (5.9)	3 (8.6)
Time between surgery and recurrent radicular pain (days), median (IQR)	77.0 (46.0–142.0)	99.0 (91.0–153.0)	92.0 (61.0–153.0)
Time between last surgery and inclusion (years), median (IQR)	1.9 (1.4–3.0)	2.7 (1.8–4.5)	2.3 (1.6–3.6)
Co-interventions, n (%)	15 (83.3)	15 (88.2)	30 (85.7)
Analgesics	14 (77.8)	15 (88.2)	29 (82.9)
Grade 1	9 (50.0)	8 (47.1)	17 (48.6)
Grade 2	12 (66.7)	11 (64.7)	23 (65.7)
Grade 3	2 (11.1)	1 (5.9)	3 (8.6)
Nonsteroidal anti-inflammatory drugs	5 (27.8)	5 (29.4)	10 (28.6)
Corticosteroids	2 (11.1)	2 (11.8)	4 (11.4)
Antidepressants	9 (50.0)	11 (64.7)	20 (57.1)
Anxiolytics	3 (16.7)	3 (17.6)	6 (17.1)
Antiepileptics	8 (44.4)	8 (47.1)	16 (45.7)
Retractile scar, n (%)	13 (72.2)	10 (58.8)	23 (65.7)
Nerve root enhancement, n (%)	14 (77.8)	11 (64.7)	25 (71.4)
Nerve root enlargement, n (%)	11 (61.1)	4 (23.5)	15 (42.9)
Arachnoiditis, n (%)	3 (16.7)	2 (11.8)	5 (14.3)
Modic 1 changes, n (%)	10 (55.6)	9 (52.9)	19 (54.3)

*BMI* Body mass index, *FAB-Q* Fear Avoidance Beliefs Questionnaire, *HAD-S* Hospital Anxiety Depression Scale, *IFX* Infliximab, *IQR* Interquartile range, *MRI* Magnetic resonance, imaging, *N* Absolute frequency, *VAS* Visual analog scale

### Primary outcome

The placebo and IFX group did not differ in the primary outcome: at day 10, three (17.6 %) versus five (27.8 %) patients showed a 50 % reduction in sciatica pain (*p* = 0.69; Table 2).

**Table 2:** Change in radicular pain at days 0 and 10 after injection

Outcomes	IFX group	Placebo group	All patients	*p*-value
	n = 18	n = 17	n = 35	
Day 0 after injection				
Absolute VAS change, median (IQR)	−26.8 (–54.8 to –14.3)	−56.7 (–73.3 to –28.6)	−36.0 (–72.3 to –14.3)	0.22
50 % reduction in sciatica pain, n (%)	5 (27.8)	9 (52.9)	14 (40.0)	0.13
PASS <40 mm, n (%)	5 (27.8)	12 (70.6)	17 (48.6)	0.01*
MCII ≥15 mm, n (%)	12 (66.7)	13 (76.5)	25 (71.4)	0.52
MCII ≥20 %, n (%)	11 (61.1)	13 (76.5)	24 (68.6)	0.33
Day 10 after injection				
Absolute VAS change, median (IQR)	−14.9 (–50.0 to 3.3)	0.0 (–30.9 to 10.0)	−9.2 (–38.5 to 9.1)	0.21
50 % reduction in sciatica pain, n (%)	5 (27.8)	3 (17.6)	8 (22.9)	0.69
PASS <40 mm, n (%)	4 (22.2)	4 (23.5)	8 (22.9)	0.94
MCII ≥15 mm, n (%)	9 (50.0)	5 (29.4)	14 (40.0)	0.21
MCII ≥20 %, n (%)	8 (44.4)	5 (29.4)	13 (37.1)	0.36

*p <0.05 comparing IFX and placebo groups by Wilcoxon Mann-Whitney test for quantitative variables and Fisher’s exact test for qualitative variables

*IFX* Infliximab, *IQR* Interquartile range, *MCII* Minimum Clinically Important Improvement, *n* Absolute frequency, *PASS* Patient Acceptable Symptom State, *VAS* Visual analog scale

### Secondary outcomes

**Fig. 2:**
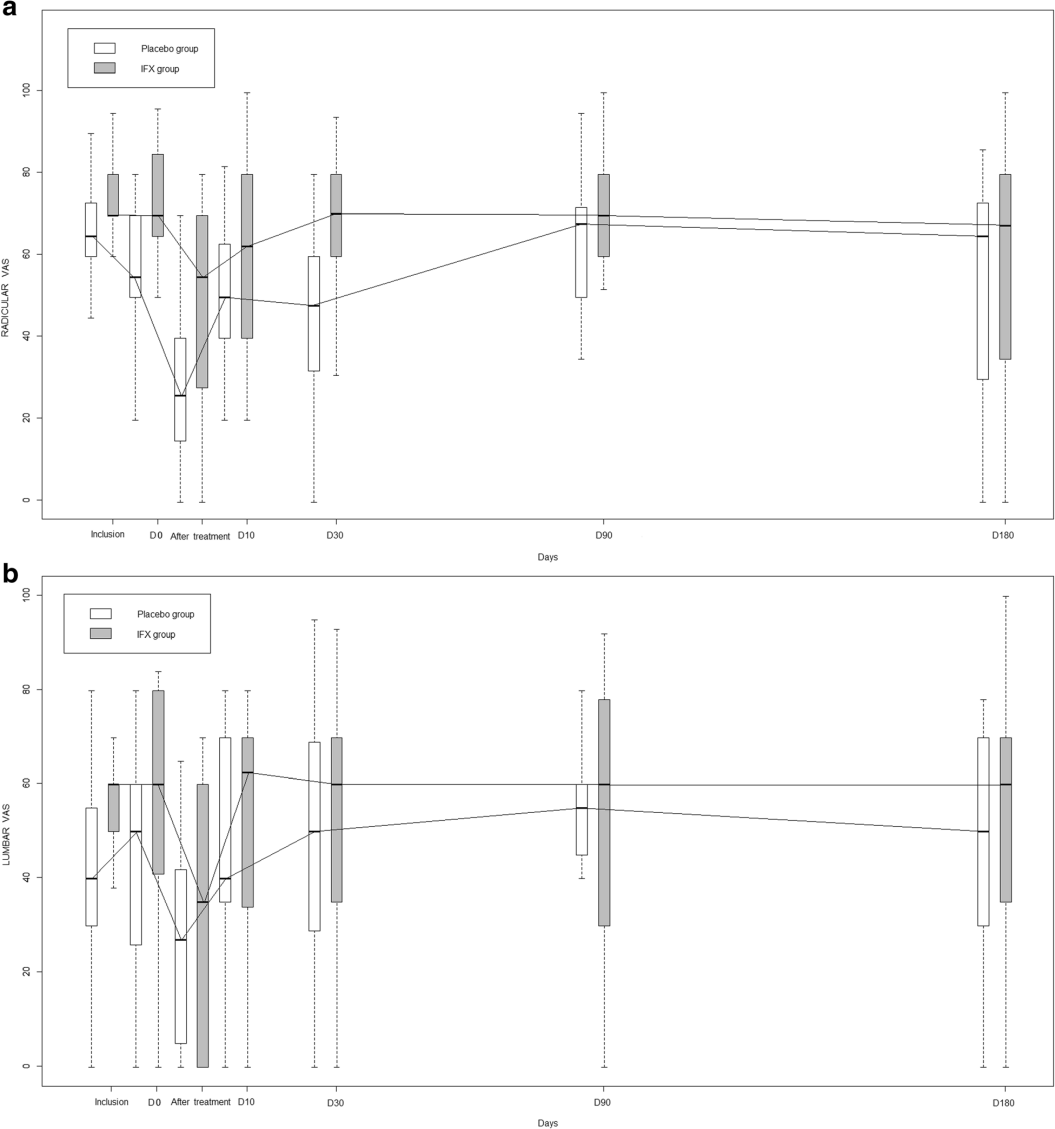
Changes in **a** radicular and **b** lumbar pain scores measured on a visual analog scale over time in patients receiving infliximab and placebo. Box-and-whisker plots represent results expressed in median and interquartile range

In the intent-to-treat analysis, between baseline and 10 days, the median (IQR) absolute change in radicular VAS pain score in the placebo and IFX group was 0.0 (–30.9 to –10.0) and –14.9 (–50.0 to –3.3) mm, respectively (*p* = 0.21) and the median absolute change in lumbar VAS pain score was 16.7 (–16.7 to –34.6) and –6.0 (–20.0 to –2.9) mm, respectively (Fig. 2). When adjusting for baseline VAS radicular pain score, the ANCOVA analysis did not reveal any treatment effect (data not shown). The two groups did not differ in all other secondary outcomes, except for number of patients reaching the PASS for radicular pain (VAS <40 mm), which was significantly higher for the placebo than IFX group at day 0 after injection (12 (70.6 %) versus five (27.8 %); *p* = 0.01; Table 2). Overall, radicular and lumbar pain and disability scores remained stable over time in both groups (Tables 2 and 3). No drug-sparing effect was observed in the IFX group compared to the placebo group, whatever the co-intervention assessed (Appendix 1).

**Table 3:** Radicular and lumbar pain scores and Québec disability score during follow-up

Time points	IFX group	Placebo group	All patients
	n = 18	n = 17	n = 35
Radicular pain, VAS score (0–100 mm)			
Day 0	70.0 (65.0–85.0)*	55.0 (50.0–70.0)	70.0 (55.0–80.0)
Day 0 post-injection	55.0 (28.0–70.0)	26.0 (15.0–40.0)	40.0 (18.0–60.0)
Day 10	62.5 (40.0–80.0)	50.0 (40.0–63.0)	58.0 (40.0–75.0)
Day 30	70.5 (60.0–80.0)	48.0 (32.0–60.0)	60.0 (40.0–75.0)
Day 90	70.0 (60.0–80.0)	68.0 (50.0–72.0)	68.0 (52.0–78.0)
Day 180	67.5 (45.0–80.0)	65.0 (30.0–73.0)	65.0 (35.0–80.0)
Lumbar pain, VAS score (0–100 mm)			
Day 0	60.0 (41.0–80.0)	50.0 (26.0–60.0)	55.0 (35.0–70.0)
Day 0 post-injection	35.0 (0.0–60.0)	27.0 (5.0–42.0)	30.0 (0.0–60.0)
Day 10	62.5 (34.0–70.0)	40.0 (35.0–70.0)	60.0 (34.0–70.0)
Day 30	60.0 (35.0–70.0)	55.0 (30.0–69.0)	60.0 (30.0–70.0)
Day 90	60.0 (30.0–78.0)	55.0 (45.0–60.0)	55.0 (40.0–70.0)
Day 180	60.0 (35.0–70.0)	50.0 (30.0–70.0)	60.0 (32.0–70.0)
Québec disability score (0–100)			
Day 0	55.0 (37.0–68.0)	45.0 (31.0–62.0)	51.0 (37.0–65.0)
Day 10	51.5 (39.0–66.0)	48.0 (39.0–55.0)	51.0 (39.0–59.0)
Day 30	57.5 (38.0–67.0)	45.0 (33.0–55.0)	47.0 (34.0–66.0)
Day 90	53.5 (38.0–69.0)	44.5 (36.0–53.5)	46.5 (38.0–64.0)
Day 180	53.5 (37.0–70.0)	44.0 (34.0–51.0)	48.0 (34.0–60.0)

Data are median (interquartile range)

*IFX* Infliximab, *VAS* Visual analog scale

*p<0.05 compared to placebo group

### Safety

Overall, 128 adverse events were reported, 65 in the placebo group and 63 in the IFX group, but none was considered serious or related to treatment. The most frequently reported adverse events were increased radicular pain, infections, gastrointestinal symptoms, joint pain and respiratory symptoms, and were observed in 51.4 %, 80.0 %, 40.0 %, 34.3 %, and 20.0 % of patients, respectively, with no significant difference between the two treatment groups (Appendix 2).

## Discussion

TNF-α could be a key pro-inflammatory and pro-fibrotic cytokine in the genesis of post-operative peridural fibrosis and subsequent painful symptoms. In this double-blind, randomized controlled trial, we assessed the efficacy and safety of TNF-α blockade with a single intravenous injection of IFX, 3 mg/kg, in managing sciatica pain due to post-operative peridural lumbar fibrosis. A single 3-mg/kg IFX injection had no clinically significant effect on sciatica pain as compared with placebo at day 10. The number of patients reaching the PASS for radicular pain was significantly higher in the placebo than IFX group at 2 hours after injection. In addition, ANCOVA revealed that the changes in radicular VAS pain at days 0 and 10 after injection were explained by radicular VAS pain at baseline rather than a treatment effect (estimated coefficient 0.77 (95 % confidence interval 0.25–1.30), *p* = 0.01, and 0.87 (0.40–1.34), *p* < 0.001, respectively; data not shown). Overall, the treatment was well tolerated, and no serious adverse events related to the treatment occurred during the study period.

Several hypotheses could explain the lack of efficacy of a potent active treatment in a randomized controlled trial, including a high placebo effect or spontaneous improvement resulting in sustained beneficial effects in all treatment groups, the design of the study, insufficient dose and the lack of efficacy of the drug or the importance of imbalance of baseline VAS.

Intravenous injection is commonly associated with a powerful placebo effect that can persist for months or even years and limits the ability to detect effects specific to the treatment [42]. Consistent with previous reports of an early placebo effect of saline infusion on sciatica pain [43, 44], our study showed a powerful immediate placebo effect, as suggested by the early and strong improvement in radicular and lumbar pain scores as well as a higher rate of patients reaching the PASS in the placebo than IFX group at 2 hours after injection. Korhonen et al*.* assessed the efficacy of IFX compared to saline in sciatica by disk herniation; approximately 15 % of the patients in the saline group had an immediate reduction of at least 75 % of sciatica pain at day 0 [43, 44]. In our study, the immediate placebo effect may have been greater in the saline group than in the IFX group. However, determinants of this early placebo effect of intravenous injections have not been clearly identified yet. At day 10, we did not observe a high placebo response that may have skewed the results for our primary outcome: the median (IQR) absolute change in radicular VAS pain score for the placebo and IFX group was 0.0 (–30.9 to 10.0) and –14.9 (–50.0 to 3.3) mm, respectively (*p* = 0.21). Contrary to what has been previously reported in acute or subacute disk herniation-induced sciatica pain [45], we found no significant spontaneous improvement over time for leg pain, back pain or disability, which therefore did not interfere with the treatment effect. The stability of symptoms over the 180-day follow-up is most likely explained by the chronicity of the fibrotic changes with a median (IQR) duration time between last lumbar surgery and inclusion of 2.3 (1.6–3.6) years and by the severity of the condition, with high levels of disability and failure of previous treatments. Moreover, fibrotic processes encompass various stages. In the earliest stages, local inflammation is thought to play an important role but might not be important later on. Therefore, there might be a therapeutic window of opportunity for anti-inflammatory treatment in the fibrotic process.

For the intervention group, IFX may not have been administered at the optimal therapeutic dose or the exposure duration to the drug may not have been long enough. The dosing regimen we used was based on previous published studies addressing the efficacy of intravenous IFX for treatment of acute or subacute disk herniation-induced sciatica pain [46] designed with a single injection of 3 mg/kg [47, 48] or 5 mg/kg IFX [43, 44]. This design might not have been appropriate for a more chronic inflammatory condition such as peridural fibrosis. Indeed, in chronic inflammatory conditions, such as rheumatoid arthritis, a dose optimization is recommended every 8 weeks, starting from 3 mg/kg [49]. However, in the absence of previously published data, and considering the balance of risks and benefits of TNF-α blockade, we did not optimize the IFX dose and used a single injection at the lower dose. Furthermore, despite some experimental and clinical evidence, targeting TNF-α in late-stage fibrotic processes might not be efficient. Only small studies assessed the efficacy of IFX in fibrosis-associated human diseases including primary sclerosing cholangitis and pulmonary fibrosis associated with collagen vascular disease and failed to demonstrate a clear benefit [50, 51].

In our study, the prevalence of Modic 1 vertebral endplate subchondral bone changes was high in both groups (55.6 % in the IFX group versus 52.9 % in the placebo group). Modic 1 changes have been reported to be biomarkers of a subset of nonspecific chronic low back pain patients who display a particular clinical, biological and MRI phenotype, leading to the concept of ‘active discopathy’ [52]. The role of local inflammation in the pathogenesis of active discopathy has been suggested, with studies reporting the efficacy of local treatment with intradiscal anti-inflammatory drugs on lumbar pain [53, 54]. However, we did not observe any differences between the two groups on lumbar pain. Hypotheses to explain the lack of efficacy of anti-TNF-α therapy on lumbar pain are the same as above. In addition, one can hypothesize that TNF-α might not be central in generating active discopathy-related Modic 1 signal and back pain symptoms, as suggested by animal models [55, 56].

Some factors may limit the generalizability of our findings. Selecting inclusion and exclusion criteria is challenging in chronic pain studies. The refractoriness to treatment among patients with a longer duration of symptoms and significant coexisting psychopathology is likely to result in lower response rates for treatment participants [57, 58]. However, in our study, clinically significant symptoms of anxiety and depression assessed by the HAD-S, fear-avoidance beliefs by the FAB-Q and coping strategies by the Coping Strategies Questionnaire (data not shown) were comparable between the two treatment groups over time. Finally, one of the major challenges of this trial was to enroll a sufficient number of patients. One can hypothesize that the latest improvements in surgical techniques in the past decade have led to a reduction in the extent of peridural fibrotic scar, and to a decreased prevalence of failed back surgery syndrome associated with this condition.

## Conclusions

This randomized controlled trial did not demonstrate a clinically significant effect of a single intravenous injection of IFX at 3 mg/kg. However, from our knowledge of the pathogenesis of pain, targeting local chronic inflammation and fibrotic processes closely related to painful symptoms might still be relevant for managing this condition. We cannot conclude whether a higher dose or longer or earlier exposure to the drug would have provided more benefit, and therefore we cannot recommend the clinical use of TNF-α blockade for recurrent chronic sciatica pain associated with peridural fibrosis. Further studies are warranted to confirm our results and to address the effects of other anti-inflammatory or anti-fibrotic agents with dose optimization.

## Appendix 1

ᅟ

**Table 4:** Co-interventions during follow-up

Timepoints	IFX Group n=18	Placebo group n=17	Total n=35
	Analgesics		
Day 0	14 (77.8)	15 (88.2)	29 (82.9)
Day 10	14 (77.9)	16 (94.1)	30 (85.7)
Day 30	15 (83.3)	16 (94.1)	31 (88.6)
Day 90	14 (77.8)	16 (94.1)	30 (85.7)
Day 180	15 (83.3)	15 (88.2)	30 (85.7)
	Non-steroidal anti-inflammatory drugs		
Day 0	5 (27.8)	5 (29.4)	10 (28.6)
Day 10	5 (27.8)	7 (41.2)	12 (34.3)
Day 30	5 (27.8	6 (35.3)	11 (31.4)
Day 90	6 (33.3)	6 (35.3)	12 (34.3)
Day 180	5 (27.8)	5 (29.4)	10 (28.6)
	Corticosteroids		
Day 0	2 (11.1)	2 (11.8)	4 (11.4)
Day 10	2 (11.1)	2 (11.8)	4 (11.4)
Day 30	2 (11.1)	2 (11.8)	4 (11.4)
Day 90	2 (11.1)	2 (11.8)	4 (11.4)
Day 180	2 (11.1)	2 (11.8)	4 (11.4)
	Antidepressants		
Day 0	9 (50.0)	11 (64.7)	20 (57.1)
Day 10	11 (61.1)	12 (70.6)	23 (65.7)
Day 30	10 (55.6)	12 (70.6)	22 (62.9)
Day 90	10 (55.6)	12 (70.6)	22 (62.9)
Day 180	9 (50.0)	11 (64.7)	20 (57.1)
	Anxiolytics		
Day 0	3 (16.7)	3 (17.6)	6 (17.1)
Day 10	3 (16.7)	4 (23.5)	7 (20.0)
Day 30	3 (16.7)	5 (29.4)	8 (22.9)
Day 90	3 (16.7)	4 (23.5)	7 (20.0)
Day 180	3 (16.7)	3 (17.6)	6 (17.1)
	Antiepileptics		
Day 0	8 (44.4)	8 (47.1)	16 (45.7)
Day 10	10 (55.6)	11 (64.7)	21 (60.0)
Day 30	10 (55.6)	10 (58.8)	20 (57.1)
Day 90	9 (50.0)	9 (52.9)	18 (51.4)
Day 180	8 (44.4)	8 (47.1)	16 (45.7)

Data are absolute frequencies (%).

## Appendix 2

ᅟ

**Table 5:** Adverse events recorded < 10 and ≥ 10 days during follow-up.

Adverse events	< 10 days		≥ 10 days		Total		
	IFX	PBO	IFX	PBO	IFX	PBO	p-value
Increased neuropathic pain	0 (0)	0 (0)	0 (0)	1 (5.9)	0 (0)	1 (5.9)	-
Increased lumbar pain	0 (0)	1 (5.9)	0 (0)	2 (11.8)	0 (0)	4 (23.5)	-
Increased radicular pain	5 (27.8)	4 (23.5)	3 (16.7)	2 (11.8)	10 (55.6)	8 (47.1)	0.56
Headache	3 (16.7)	1 (5.9)	2 (11.1)	1 (5.9)	5 (27.8)	2 (11.8)	-
Fatigue	2 (11.1)	0 (0)	2 (11.1)	1 (5.9)	5 (27.8)	1 (5.9)	-
Hospitalization	0 (0)	0 (0)	0 (0)	2 (11.8)	0 (0)	2 (11.8)	-
Hospitalization for lumbo-radicular pain	0 (0)	0 (0)	3 (16.7)	1 (5.9)	3 (16.7)	1 (5.9)	-
Infections	4 (22.2)	1 (5.9)	11 (56.0)	13 (76.4)	14 (77.8)	14 (82.4)	0.93
Cardiovascular symptoms	0 (0)	1 (5.9)	0 (0)	1 (5.9)	0 (0)	2 (11.8)	-
Skin symptoms	1 (5.6)	1 (5.9)	1 (5.6)	2 (11.8)	2 (11.1)	3 (17.6)	-
Gastrointestinal symptoms	2 (11.1)	2 (11.8)	6 (33.3)	4 (23.5)	8 (44.4)	6 (35.3)	0.53
Gynecological symptoms	0 (0)	0 (0)	0 (0)	2 (11.8)	0 (0)	2 (11.8)	-
Hormonal manifestations	2 (11.1)	2 (11.8)	0 (0)	0 (0)	2 (11.1)	2 (11.8)	-
Joint pain	2 (11.1)	0 (0)	4 (22.2)	6 (35.3)	6 (33.3)	6 (35.3)	0.95
Psychiatric disorder	1 (5.6)	0 (0)	0 (0)	1 (5.9)	1 (5.6)	1 (5.9)	-
Respiratory symptoms	0 (0)	1 (5.9)	2 (11.1)	4 (23.5)	2 (11.1)	5 (29.4)	-
Sleeping disorder	0 (0)	0 (0)	1 (5.6)	1 (5.9)	1 (5.6)	1 (5.9)	-
Sexual disorder	0 (0)	1 (5.9)	1 5.6)	1 (5.9)	1 (5.6)	3 (17.6)	-
Traumatism	0 (0)	1 (5.9)	0 (0)	0 (0)	0 (0)	1 (5.9)	-
Vertigo	0 (0)	0 (0)	3 (16.7)	0 (0)	3 (16.7)	0 (0)	-
Total	22	17	38	44	63	65	-

IFX: IFX group; PBO: placebo group.

Data are absolute frequencies (%).

## Abbreviations

ANCOVA: Analysis of covariance
FAB-Q: Fear-Avoidance Beliefs Questionnaire
HAD-S: Hospital Anxiety and Depression Scale
IFX: Infliximab
IQR: Interquartile range
MCII: minimum clinically important improvement
MRI: Magnetic resonance imaging
PASS: Patient acceptable symptom state
TNF: Tumor necrosis factor
VAS: Visual analog scale
